# Relative longevity among retired military personnel: a historical-cohort study

**DOI:** 10.1186/s40779-015-0057-y

**Published:** 2015-10-30

**Authors:** Michael Hartal, Yitshak Kreiss, Nirit Yavnai

**Affiliations:** Israel Defense Forces Medical Corps, Military PO Box 02149, Tel Hashomer, Israel; Department of Military Medicine, Hebrew University Faculty of Medicine, Jerusalem, Israel

**Keywords:** Occupation, Life expectancy, Longevity, Military personnel

## Abstract

**Background:**

Occupation is a significant factor affecting life, health and well-being. Long-term military service is a unique career path that may have an influence on life expectancy, even after excluding obvious risks such as battlefield mortality. However, it remains unclear what the effects of a military career are on the life trajectory of personnel after retiring from service. This study compared life expectancy among retired military personnel (RMP) to their sex and birth cohort-specific reference populations.

**Methods:**

For this historical cohort study, we collected data on the sex, year of birth, year of death, time in service, and rank at end of service for 4862 Israeli RMPs. Data on reference populations were provided by the Israel Central Bureau of Statistics by birth decade from 1900 to 1989. We calculated the difference between each individual RMP’s age at death and the “expected” age at death, based on sex and birth cohort-specific means in the reference populations.

**Results:**

Overall, 67.9 % of RMPs lived longer than average relative to their sex-specific birth cohort. This difference in life expectancy was more pronounced among women than among men. There was a significant trend of increasing differences between RMP males and reference males over time (*P* < 0.002), whereas no significant trend was identified among females. Length of service and rank were not associated with relative longevity for RMPs.

**Conclusions:**

The mechanism of the protective effect of military service on life expectancy remains unknown, but our findings indicate that it affects men and women differently, with women being more likely to benefit from the potential protective effect of military service. The healthy worker effect is known to vary from one occupation to another, and to the best of our knowledge, this is the first attempt to quantify the magnitude of the healthy worker effect among career military servicemen and women.

## Background

Occupation is a significant factor affecting life, health and well-being. Several factors likely contribute to this situation, including income, environmental conditions and personal satisfaction and fulfillment [1, 2]. The study of associations between life parameters and health outcomes is a basic tenet of epidemiological research, and for two centuries occupational factors have been linked to patterns of mortality [3, 4]. Despite the considerable changes to job characteristics that have taken place over the past 50 years, few changes in mortality patterns have been seen across social groups [4]. In an analysis of modern occupations, one recent study found that younger age at death was observed for certain job categories such as athletes and performers, while older age at death was seen for other groups, such as political and business workers [3].

While the association between occupation and life expectancy may be complicated by self-selection, demographic and socioeconomic characteristics may affect opportunities for entry into certain careers. There is also evidence that occupation may causally influence life expectancy. For example, for black and white athletes a career in professional sports was found to substantially narrow the typical racial gap in life expectancy, a phenomenon attributed to the unique characteristics of this occupation [5].

Long-term military service is another unique career path that may influence basic health outcomes such as life expectancy. After excluding obvious reasons for shortened life expectancy, such as battlefield injury and death, it is unclear what the effects of a military career are on the life trajectory of personnel once they retire from service. On one hand, some researchers have suggested that military service may serve as a protective factor for life course events such as education, family, earnings and post-service employment [6–8]. Despite the erratic work life associated with a military career, veterans are able to leverage their challenging vocation to equal the occupational achievements of nonveterans. Additionally, personnel starting their military careers at an early age showed a larger gain in psychological strength and health from adolescence through mid-life as compared to nonveterans [9]. Furthermore, an older-than-average age at death has been observed among those who served in the US military [3].

On the other hand, military personnel, on average, spend less time with their families, have spouses who are less likely to work, and experience higher divorce rates [10]. Some research also indicates that veterans may be disadvantaged with regards to their ability to achieve a desired life trajectory, compared to civilians of similar background and age [6, 8].

The aim of this historical-cohort study was to compare life expectancy among retired military personnel (RMP) to their sex and birth cohort-specific reference populations.

## Methods

Data on military personnel were provided by the Israel Defense Forces (IDF) veterans’ organization and included sex, date of birth, date of death, time in service, and rank at end of service. The veterans’ organization maintains ongoing contact and communication with RMPs, up to and including notification of death, at which time insurance benefits are paid to surviving family members. Thus, these data were deemed complete and reliable. Data on medical screening procedures prior to recruitment were not available. Rank was categorized as junior commissioned officer (lieutenant [NATO equivalent OF-1] through major [OF-3]), senior commissioned officer (lieutenant colonel [OF-4] through lieutenant general [OF-8]), and noncommissioned officers (sergeant [OR-5] through chief warrant officer [OR-9]).

Data on mean age at death in the reference population, stratified by sex and birth cohort, were provided by the Israel Central Bureau of Statistics. To limit the reference populations to those most appropriate for comparison to the RMPs, we excluded non-Jewish persons, individuals who died prior to the age of 20 and immigrants who completed fewer than 10 years of life in Israel before their death.

RMPs and their reference populations were categorized by birth decades from 1900 to 1989. On initial descriptive analysis of the data, we discovered no deceased RMPs in the 1980–1989 birth cohort and only one RMP in the 1970–1979 birth cohort who had died by the time of the study. Thus, we limited our final analysis to the seven birth cohorts between 1900 and 1969.

We conducted univariable and multivariable analyses to evaluate the association between military service and age at death. To facilitate this comparison, we calculated the difference between each individual RMP’s age at death and the “expected” age at death, based on sex and birth cohort-specific means in the general population. For logistic regression analysis, we included the predictor variables sex, rank, length of military service and birth cohort. Rank and length of service were introduced into the model as dichotomous variables (commissioned officer vs. noncommissioned officer, ≤20 years vs. >20 years), as these categorizations were found to provide the best goodness of fit and prevented collinearity. The outcome variable was defined as older or younger than the expected reference value. Variables with *P* values <0.1 on univariable analysis were included in the multivariable model. The Mann Kendall test for trend was used to examine age differences over time. Statistical analysis was carried out using IBM SPSS version 21.

## Results

The characteristics of the RMPs included in the study (*n* = 4862) are presented in Table 1. This population was predominantly male, and nearly two-thirds were commissioned officers. The largest birth cohort was born in the 1920s, and 87.9 % of the RMPs were born between 1910 and 1949. The majority of deaths (84.6 %) occurred between 1990 and 2012. The mean age at retirement was 46.48 ± 6.63 years (median 45.31), and the mean age at death was 69.40 ± 12.55 years (median 69.85).

**Table 1:** Characteristics of study population (*n*=4862)

Variable	Category	*n*
Gender	Male	4318 (88.8%)
	Female	544 (11.2%)
Rank	Junior commissioned officer	1359 (28.5%)
	Senior commissioned officer	1775 (37.0%)
	Noncommissioned officer	1654 (34.5%)
Birth cohort	1900–1909	131 (2.7%)
	1910–1919	671 (13.9%)
	1920–1929	1748 (36.1%)
	1930–1939	998 (20.6%)
	1940–1949	839 (17.3%)
	1950–1959	414 (8.6%)
	1960–1969	35 (0.7%)
Year of death	1960–1969	28 (0.6%)
	1970–1979	174 (3.6%)
	1980–1989	547 (11.3%)
	1990–1999	1112 (22.9%)
	2000–2012	3001 (61.7%)

Data on rank were missing for 64 subjects; for birth cohort, 26 subjects

Table 2 presents the mean age at death by sex and birth cohort for RMPs and their reference populations. As expected, mean age at time of death decreased with progressing (younger) birth cohorts both in the general population and among RMPs. No deaths were recorded among female RMPs in the last study cohort (1960–1969). Among RMPs, the mean age at death for females was consistently higher than for males, although the difference for the 1950–1959 cohort (32 female deaths) was not statistically significant. This sex difference narrowed monotonically with progressing birth cohorts, from a maximum of 16.87 years for the earliest cohort to 0.91 years for the latest cohort (Mann-Kendall test for trend *P* < 0.02). These observations were not apparent in the respective birth cohorts in the general population. Overall, the mean age at death among the reference population was also higher for females than for males, although in the birth cohort of 1900–1909, the difference was reversed (difference = −0.59 years) and for the birth cohort of 1940–1949, the difference between the sexes was not significant. The absolute values of the sex differences in each reference birth cohort were substantially lower than in the respective RMP cohorts; the trend in difference widths did not maintain a linear progression over time (P > 0.20).

**Table 2:** Mean age at death by gender and birth cohort for retired military personnel (RMPs) and reference populations

Birth cohort	Male		Female		Δ	95%CI	P value*
	Mean (SD)	*n*	Mean (SD)	*n*			
RMPs							
1900–1909	77.88 (10.07)	127	94.75 (5.91)	4	16.87	6.83–26.91	<0.001
1910–1919	78.28 (10.85)	583	89.06 (5.56)	88	10.78	8.47–13.09	<0.001
1920–1929	74.12 (10.08)	1526	81.14 (5.94)	222	7.02	5.66–8.38	<0.001
1930–1939	67.32 (8.68)	871	73.70 (5.34)	127	6.38	4.83–7.93	<0.001
1940–1949	58.47 (7.02)	777	60.50 (5.45)	62	2.03	0.24–3.82	0.026
1950–1959	52.47 (5.31)	382	53.38 (4.05)	32	0.91	−0.97–2.79	0.345
1960–1969	46.97 (3.65)	35	-	0	-	-	-
Reference							
1900–1909	79.91 (10.57)	115,951	79.32 (10.63)	110,373	−0.59	−0.68– −0.50	<0.001
1910–1919	76.52 (11.48)	137,389	79.13 (11.46)	149,155	2.61	2.53–2.70	<0.001
1920–1929	73.16 (11.61)	129,948	75.60 (10.96)	127,871	2.44	2.35–2.53	<0.001
1930–1939	65.35 (11.88)	71,343	67.02 (11.13)	54,062	1.67	1.54–1.80	<0.001
1940–1949	53.99 (13.09)	37,372	54.12 (12.00)	23,077	0.13	−0.08–0.34	0.22
1950–1959	44.91 (12.00)	23,347	47.48 (10.05)	13,330	2.57	2.33–2.81	<0.001
1960–1969	36.56 (9.16)	9,056	38.35 (8.42)	4,687	1.79	1.48–2.10	<0.001

Δ = Difference between the means

*Independent samples *t*-test

Table 3 shows the mean age at death by study group and birth cohort, separately for males and females. Among both sexes, and among all birth cohorts (other than males born in 1900–1909), the mean age at death was significantly higher among RMPs than among their civilian counterparts. The width of this occupational group difference was more pronounced among women (weighted mean difference 9.37 years, maximum difference 15.43 years) than among men (weighted mean difference 1.35 years, maximum difference 10.41 years). Differences in mean age at death between RMP males and reference males increased significantly over time (*P* < 0.002), whereas no significant trend was identified among females.

**Table 3:** Mean age at death for retired military personnel (RMPs) and their reference populations, stratified by gender and birth cohort

Birth cohort	RMPs		Reference		Δ	95%CI	P value*
	Mean (SD)	*n*	Mean (SD)	*n*			
Male							
1900–1909	77.88 (10.07)	127	79.91 (10.57)	115951	−2.03	−3.874– −0.186	0.0305
1910–1919	78.28 (10.85)	583	76.52 (11.48)	137,389	1.76	0.824–2.696	<0.001
1920–1929	74.12 (10.08)	1526	73.16 (11.61)	129,948	0.96	0.374–1.547	0.001
1930–1939	67.32 (8.68)	871	65.35 (11.88)	71,343	1.97	1.177–2.763	<0.001
1940–1949	58.47 (7.02)	777	53.99 (13.09)	37,372	4.48	3.555–5.405	<0.001
1950–1959	52.47 (5.31)	382	44.91 (12.00)	23,347	7.56	6.352–8.768	<0.001
1960–1969	46.97 (3.65)	35	36.56 (9.16)	9,056	10.41	7.367–13.453	<0.001
Female							
1900–1909	94.75 (5.91)	4	79.32 (10.63)	110373	15.43	4.987–25.873	0.004
1910–1919	89.06 (5.56)	88	79.13 (11.46)	149,155	9.93	7.530–12.330	<0.001
1920–1929	81.14 (5.94)	222	75.60 (10.96)	127,871	5.54	4.094–6.986	<0.001
1930–1939	73.70 (5.34)	127	67.02 (11.13)	54,062	6.68	4.739–8.621	<0.001
1940–1949	60.50 (5.45)	62	54.12 (12.00)	23,077	6.38	3.385–9.375	<0.001
1950–1959	53.38 (4.05)	32	47.48 (10.05)	13,330	5.90	2.409–9.391	<0.001
1960–1969	-	0	38.35 (8.42)	4,687	-	-	-

Δ = Difference between the means

*Independent samples *t*-test

Overall, 67.9 % of RMPs lived longer than average relative to their sex-specific birth cohort. This proportion was strongly modified by sex, with 65.6 % of male RMPs outliving their civilian counterparts compared to 86.9 % of females (OR = 3.49, *P* < 0.001). There was a clear increase in the odds of relative longevity for RMPs in progressive birth cohorts with a dramatic increase for the latest birth decades (Table 4). Length of service and rank were not associated with relative longevity for RMPs and thus were not included in the multivariable model. Table 5 presents the results of the multivariable regression model, which remained largely similar to univariable results. After adjusting for cohort effect, female sex was an independent predictor of RMP longevity relative to the reference population (OR = 3.83, 95%CI 2.95–4.98), with the odds of relative longevity among progressive RMP birth cohorts largely unchanged compared to the univariable analysis.

**Table 4:** Univariable logistic regression for relative longevity of retired military personnel

Variable	Category	OR	95%CI	P value
Gender	Male	1.00		<0.001
	Female	3.49	2.69–4.52	
Rank	Noncommissioned officer	1.00		0.134
	Commissioned officer	0.91	0.80–1.03	
Length of service	≤20 years	1.00		0.186
	>20 years	0.85	0.67–1.08	
Birth cohort	1900–1909	1.00		
	1910–1919	2.26	1.55–3.30	<0.001
	1920–1929	2.09	1.46–2.98	<0.001
	1930–1939	2.55	1.76–3.69	<0.001
	1940–1949	3.72	2.55–5.43	<0.001
	1950–1959	13.22	8.13–21.48	<0.001
	1960–1969	20.77	4.78–90.17	<0.001

**Table 5:** Multivariable logistic regression for relative longevity of retired military personnel

Variable	Category	OR	95%CI	P value
Gender	Male	1.00		
	Female	3.83	2.95–4.98	<0.001
Birth cohort	1900–1909	1.00		
	1910–1919	2.046	1.40–3.00	<0.001
	1920–1929	1.891	1.32–2.71	<0.001
	1930–1939	2.324	1.60–3.37	<0.001
	1940–1949	3.611	2.47–5.28	<0.001
	1950–1959	12.900	7.92–21.00	<0.001
	1960–1969	21.588	4.97–93.78	<0.001

## Discussion

In this study, we found that, on average, RMPs outlived the general population. Females outlived males in both RMPs and the general population, but the characteristics of this sex differential varied greatly between RMPs and their referent groups. Among RMPs, the absolute difference between females and males increased monotonically with increasing age (earlier cohorts), while among the reference population, the sex differences were generally stable and of a smaller magnitude. The fact that this sex difference narrowed over time in RMPs but not in the general population seems to indicate that this is a phenomenon unique to military careers.

The difference in longevity between RMPs and their civilian reference populations was pronounced in young males (later cohorts) but diminished among older men (earlier cohorts). In contrast, the difference in females was prolonged and remained stable into older age. The mechanism of the protective effect of military service on life expectancy is unknown, but this finding seems to indicate that it affects men and women differently, providing shorter-term protection for males than for females. Alternatively, military service may provide better protection against causes of death that are more frequent among females than among males. However, this latter point could not be verified with the available data as this study was not designed to investigate specific causes of death.

Younger military retirees had the greatest odds of outliving their civilian counterparts. These increased odds of longevity extended into older age but were substantially reduced with increasing age. This observation remained unchanged after adjusting for any changes in the male–female composition of the military over time. Thus, both sex and birth cohort remained independent predictors of relative longevity in RMPs relative to the general population.

Females were more likely than males to benefit from the protective effect of military service. Because the recruitment screening process is identical for males and females, differential health selection of females is not a plausible explanation for this observation. Given that women generally outlive men, the phenomenon of a substantially longer life expectancy observed among female RMPs in this study may possibly be due to the military selection process, which favors healthy individuals. This selection process may enhance and amplify the expression of any anticipated life expectancy differences between sexes in this unique occupational group [11].

Interestingly, neither rank nor long-term military service was associated with outliving civilian counterparts. This finding might indicate either that the protective effect of military service occurs in the earlier stages of a military career or that it is the result of a selective process, such as the medical, mental and cognitive screening inherent in recruitment procedures.

The main results of this study are likely attributable to the healthy worker effect, first described some 150 years ago [12], in which people able to attend work – especially the demanding work inherent to a military environment – are necessarily healthier compared to the general population, which includes individuals too sick to work [11, 13]. The healthy worker effect is known to vary from one occupational cohort to another [11] and may possibly be accentuated in the military due to entry requirements, periodic health assessments and medical discharge from service for personnel unfit for service. To the best of our knowledge, this is the first attempt to quantify the magnitude of the healthy worker effect for career military servicemen and women.

## Limitations

We compared the mean age at death among military personnel to that of the Jewish population in Israel, excluding non-Jewish minorities such as Arabs and Druze. For females, there is no question of validity in the comparison, as nearly no non-Jewish females serve in the IDF. As for males, the vast majority of RMPs are Jewish, with nearly no Arabs and only a small proportion of Druze in service. We initially used a weighted standard population average to adjust the mean age at death of the overall population for the differences in life expectancy between Jewish and Druze males. This difference, while evident among earlier birth cohorts, was negligible among birth cohorts born after 1940. Due to the very small proportion of Druze in the overall Israeli population, the weighted averages were identical to those of the Jewish population, varying by no more than ±0.01 years. Thus, we adopted the simpler, more intuitive and identical population data over a weighted, calculated and indirect adjusted population average for purposes of comparison. In sensitivity analysis, no differences were found in our results.

While our study design (inclusion criteria, exclusion criteria and stratified analyses) precluded potential confounding effects by sex, religion, untimely deaths, immigration status and the cohort effect, we were unable to adjust for income level. Additionally, this study was limited to all-cause mortality, while future studies might investigate specific causes of death and their effects on the population of RMPs. Furthermore, we did not study the sex-specific attributes of additional “exposure” variables, such as the utilization of health care services, periodic health screening, medical discharge and working patterns during and after military service. Future studies might examine the effects of these characteristics on differential life expectancy.

## Conclusion

In this large historical cohort study of nearly 5000 career military personnel and over 1 million civilians, retired soldiers, sailors, airmen and airwomen were shown to outlive their peers in the general population. The magnitude of this benefit varied by sex and by birth cohort. Future studies should examine the differential effects of military service on specific causes of death in order to better understand the mechanisms of this occupational observation.

## References

[1] Deaton A (2008). Income, health, and well-being around the world: evidence from the Gallup World Poll. J Econ Perspect.

[2] Fischer HD (1985). State of health and stress factors in occupation: the mass media profession. Soc Sci Med.

[3] Epstein CR, Epstein RJ (2013). Death in The New York Times: the price of fame is a faster flame. QJM.

[4] Lynge E (2011). Occupational mortality. Scand J Public Health.

[5] Lawler T, Lawler F, Gibson J, Murray R (2012). Does the African-American-white mortality gap persist after playing professional basketball? A 59-year historical cohort study. Ann Epidemiol.

[6] Smith-Osborne A (2013). Veterans Administration health care policies as a protective mechanism supporting an expected life trajectory after military service. Soc Work Public Health.

[7] Angrist JD (1993). The effect of veterans benefits on education and earnings. Ind Labor Relat Rev.

[8] Angrist JD (1998). Estimating the labor market impact of voluntary military service using social security data on military applicants. Econometrica.

[9] Elder GH (1986). Military times and turning points in men’s lives. Dev Psychol.

[10] Angrist JD, Johnson JH (2000). Effects of work-related absences on families: evidence from the Gulf War. Ind Labor Relat Rev.

[11] Baillargeon J (2001). Characteristics of the healthy worker effect. Occup Med.

[12] Heederik D (2006). Micro‐epidemiology of the healthy worker effect?. Occup Environ Med.

[13] Pearce N, Checkoway H, Kriebel D (2007). Bias in occupational epidemiology studies. Occup Environ Med.

